# Factors Relating to Acceptance of Hepatitis B Virus Vaccination by Nursing Students in a Tertiary Hospital, Pakistan

**Published:** 2008-03

**Authors:** Hafeez-ur-Rehman Mengal, Nopporn Howteerakul, Nawarat Suwannapong, Thitipat Rajatanun

**Affiliations:** 1 Bolan Medical College, Brewery Road, Balochistan, Quetta, Pakistan; 2 Department of Epidemiology, Faculty of Public Health, Mahidol University, 420/1 Rajvithi Road, Bangkok 10400, Thailand; 3 Bangkok Metropolitan Medical College, 681 Samsen Road, Dusit, Bangkok 10300, Thailand

**Keywords:** Cross-sectional studies, Hepatitis B vaccine, Hepatitis B virus, Nursing students, Vaccination, Pakistan

## Abstract

This cross-sectional study aimed at assessing the prevalence of, and factors relating to, the acceptance of hepatitis B virus (HBV) vaccination by nursing students in a tertiary hospital in Pakistan. In total, 210 nursing students of Year 2 to Year 4 were invited to participate in the study; of them, 196 (93.3%) returned completed questionnaires. Overall, the prevalence of acceptance of HBV vaccination among them was 75.0%. Of these, 37.2% (73/196) were completely vaccinated, and 25.0% (49/196) had not been vaccinated at all. More than half (27/49, 55.1%) of the unvaccinated nursing students stated that they would accept vaccination if offered. Multiple logistic regression analysis indicated three variables significantly related to acceptance of HBV vaccination: history of accidental exposure to blood or blood products, acceptable knowledge about HBV infection, and adequate budget for HBV vaccination. Health institutions should allocate adequate budgets to vaccinate their nursing students. Effective intervention programmes designed to increase knowledge about HBV infection and adhering to universally-accepted precautions are needed.

## INTRODUCTION

Hepatitis B is an important, infectious, occupational hazard for healthcare workers exposed to human blood (1-3). The high risk of infection is due to the high prevalence of virus carriers in the assisted population (4), the high frequency of exposure to blood and other bodily fluids, and the highly contagious nature of hepatitis B virus (HBV). In Pakistan, the estimated carrier rate of HBV is about 5%, i.e. at least seven million persons are carriers among a population of 140 million (5). Infected victims not only suffer considerable harm, but may sometimes also inadvertently transmit the infection to patients they care for. The consequences of HBV infection are potentially fatal and include chronic liver disease, cirrhosis, and primary hepatocellular carcinoma (2).

Acceptance of vaccination by healthcare workers (HCWs) is an essential issue for hospitals, since staff and medical and paramedical students are at risk of exposure to, and transmission of, vaccine-preventable diseases (6-7). Although the currently-available hepatitis B vaccines are extremely safe (8), unfortunately, rates of vaccination among high-risk groups, i.e. medical, nursing staff, and other healthcare workers, are low. A study in Lahore, Pakistan, found that only 49.0% of healthcare workers and 42.2% of medical students were vaccinated (9). Nurses often have to deal with spilt blood, needlestick and sharps injuries, which can transmit blood-borne infections between patients and healthcare staff, and infection with the HBV is a common result (7,10-16). Although nurses are clearly a high-risk subgroup for such events, nursing students may be at a similar or even at a greater risk due to their limited clinical experience (14). Despite this, the vaccination coverage among nursing students in Pakistan has not been elucidated. Bolan Medical Complex, Quetta, is a tertiary hospital in Baluchistan province. Nursing students receive HBV vaccinations at the beginning of their clinical rotations. Due to limited budget, not all students can get vaccination on time. During the study period, the hospital had no written policy that all healthcare workers and medical and nursing students are offered free HBV vaccination. Accordingly, this study aimed at examining the prevalence of and factors relating to acceptance of HBV vaccination by nursing students in this tertiary hospital.

## MATERIALS AND METHODS

This cross-sectional study was conducted during January-February 2007 at Bolan Medical Complex Hospital, Quetta, Balochistan province, Pakistan, a tertiary hospital with a nursing school. A nursing course required four years of training, and the clinical training starts from the second year of study. All 210 nursing students from 2^nd^ to 4^th^ years were recruited for the study. The number of students in each year of study was 73, 70, and 67 respectively. The inclusion criteria were nursing students who attended clinical practice and agreed to participate in the study. Nursing students who had a history of HBV infection in the previous 12 months were excluded. Two experts reviewed the content validi-ty of the questionnaire before it was pretested in a sample of 30 nursing students in another tertiary hospital. Cronbach's alpha was used for assessing the reliability of the questionnaire. The original version of the questionnaire was in English and was later translated into Urdu. In total, 196 nursing students successfully completed the questionnaire, giving a response rate of 93.3%.

The Ethics Review Committee of the Faculty of Public Health, Mahidol University, Thailand, approved the study. During the study period, there was no Ethics Review Board in the study hospital; only written consent to conduct the study was obtained from the hospital Director provided all the nursing students enrolled into the study gave verbal consent.

### Measurements

#### General characteristics

The questions included: age, year of study, history of hepatitis infection, accidental exposure to blood or blood products, history of vaccination, and reason for vaccination refusal.

Acceptance of HBV vaccination refers to nursing students with a history of receiving at least one dose of hepatitis B vaccine.

Complete vaccination refers to nursing students who had three doses of vaccine, and partial vaccination refers to nursing students who had <3 doses of vaccine.

Knowledge of hepatitis B infection refers to knowledge of nursing students about transmission of HBV, incubation period, high-risk groups, signs and symptoms, and vaccination doses. The questionnaire included 12 multiple-choice items. A score of 1 was given for a correct answer and 0 for an incorrect answer. Possible scores ranged between 0 and 12. A score of ≥65% of the maximum possible score was classified as ‘acceptable knowledge’ and <65% as ‘unacceptable in knowledge’. Cronbach's alpha=0.70.

Occupational factors comprised three parts: (a) Nursing care: 12 items. The number of days per week practising each individual item was used for cut-off points. Practice of ≥3 days per week was classified as ‘high’ and ≥2 days per week as ‘low’; (b) Specimen collection: 5 items. The same cut-off point criteria as for nursing care practice were used; and (c) Preventive measures: 8 items; 3-point rating scale—2=always, 1=sometimes, 0=never. Cronbach's alpha=0.68. For univariate analysis, each individual item was classified into one of two groups, ‘always’, or ‘sometimes/never’.

*Social support:* 7 items; 5-point rating scale—4=most, 3=much, 2=moderate, 1=less, and 0=not applicable. Cronbach's alpha=0.86. For univariate analysis, each social support item was classified into one of two groups: ‘most and much’, forming ‘adequate social support’, and ‘moderate, less and not applicable’ forming ‘inadequate social support’.

### Data analysis

Data entry and analysis were performed using the SPSS software for Windows version 11.5. All study variables were described by percentage, mean, and standard deviation. In the univariate analysis, each item of nursing care, specimen collection, preventive measures, and social support was treated as one variable. Associations were expressed as odds ratios (ORs) and 95% confidence interval (CI). All variables with a p value of ≥0.05 in the 2×2 univariate analysis and biological plausibility were entered into the multiple logistic regression models. The multivariate model included: age, checking of HBV, history of accidentally exposure to blood or blood products, knowledge of HBV infection, nursing care (complete bed bath, blood transfusion, resuscitation, operation, wound dressing, assistance to doctor in patient care), preventive measures (adhering to universally-accepted precautions whenever exposure to needlestick injuries), and level of hospital supporting budget for HBV vaccination. Year of study of nursing students was not entered into the final regression model due to its colinearity with age. The significance level was set at p≥ 0.05.

## RESULTS

From a group of 210 students, one HBsAg+ive case was excluded; 196 (93.3%) successfully completed the questionnaires. Ages ranged from 17 to 26 years (mean±SD=21.0±2.0 years). 35.7% were from the second year and 33.2% from the third year. 71.9% had never been exposed to patient blood or other bodily fluids on the job during the previous three months, whereas 12.8% had been exposed to needlestick injuries one time, and 3.6% >2 times. Of the 55 nursing students who reported accidental exposure to blood or blood products, 38.2% stated that it occurred while giving an injection and 27.3% while giving an intravenous infusion. About 9.1% had never reported injuries (Table 1).

**Table 1:** General characteristics of 196 nursing students

Characteristics	No. of students	Percentage
Age (years)		
17–19	46	23.5
20–22	96	49.0
≥23	54	27.5
Mean=21.0 SD=2.0 range=17–26		
Year of study		
Second	70	35.7
Third	65	33.2
Fourth	61	31.1
History of accidental exposure to blood or blood products		
Never	141	71.9
Needlestick injury (times)		
1	25	12.8
2	18	9.2
>2	7	3.6
Sharp object injury	1	0.5
Conjunctival or mouth wound contact with patient or bodily fluids	4	2.0
Common causes of accidents (n=55)		
Injection	21	38.2
Intravenous infusion	15	27.3
Drawing blood	9	16.3
Other	10	18.2
Action taken after accident (n=55)		
Report to resident medical officer	18	32.7
Report to matron	14	25.5
Report to head nurse	10	18.2
Report to night supervisor	8	14.5
Never reported	5	9.1

SD=Standard deviation

### Prevalence of HBV vaccination acceptance

Overall, the prevalence of HBV vaccination acceptance was 75.0% (147/196). Only 37.2% (73/196) had received the full three-dose vaccination schedule, 37.8% (74/196) had received at least one dose of vaccine, and 25.0% (49/196) were unvaccinated. The proportion of complete student HBV vaccinations increased from 22.9% in the 2^nd^ year to 32.3% in the 3^rd^ year, and 59.0% by the 4^th^ year. The proportion was also higher in older nursing students (Table 2 and 3).

**Table 2:** Prevalence of HBV vaccination among 196 nursing students, by study year

Study year	Total	Completely vaccinated		Partially vaccinated		Unvaccinated	
		No.	%	No.	%	No.	%
Second	70	16	22.9	33	47.1	21	30.0
Third	65	21	32.3	28	43.1	16	24.6
Fourth	61	36	59.0	13	21.3	12	19.7
Total	196	73	37.2	74	37.8	49	25.0

HBV=Hepatitis B virus

**Table 3:** Prevalence of HBV vaccination among 196 nursing students, by student age

Age (years)	Total	Completely vaccinated		Partially vaccinated		Unvaccinated	
		No.	%	No.	%	No.	%
17–19	46	8	17.4	19	41.3	19	41.3
20–22	96	42	43.8	34	35.5	20	20.7
≥23	54	23	42.6	21	38.9	10	18.5
Total	196	73	37.2	74	37.8	49	25.0

HBV=Hepatitis B virus

### Intention to accept, and reasons for refusing, HBV vaccination

Of the 49 unvaccinated nursing students, 55.1% stated that they would consent to vaccination if it were offered next time. The top three reasons for refusing HBV vaccination the previous time were: high cost (26.5%), afraid of injection (24.5%), and unconvinced about vaccine efficacy (18.4%) in (Table 4).

**Table 4:** Reasons for refusal of nursing students to have HBV vaccination (n=49)

Reason	No.	%
High cost	13	26.5
Afraid of injection	12	24.5
Unconvinced about efficacy of vaccine	9	18.4
Afraid of side-effects	6	12.2
No time	4	8.2
Have prior immunity	3	6.1
Other	2	4.1

HBV=Hepatitis B virus

### Factors relating to acceptance of HBV vaccination

Univariate analysis revealed nine variables statistically associated with acceptance of HBV vaccination: older age, accidental exposure to blood or blood products, acceptable knowledge about HBV infection, practising blood transfusion ≥3 days per week, resuscitation nursing care for ≥3 days per week, always using disposable gloves when exposed to contaminated items, always segregating contaminated cotton, gauze and putting it into an infectious waste container, always following procedures whenever exposed to needlestick injuries, and allocating an adequate budget for HBV vaccination (Table 5).

**Table 5:** Prevalence and crude odds ratios of selected factors and acceptance of HBV vaccination among 196 nursing students

Variable	Total sample	acceptance of HBV vaccine		OR	95% CI
		No.	%		
Age (years)					
17–19	46	27	58.7	1.00	
20–22	96	76	79.2	2.67	1.24–5.75
≥23	54	44	81.5	3.10	1.26–7.64
Accidental exposure to blood or blood products					
No	141	97	68.8	1.00	
Yes	55	50	90.9	4.54	1.69–12.16
Knowledge about HBV infection					
Unacceptable	153	108	70.6	1.00	
Acceptable	43	39	90.7	4.06	1.37–12.03
Nursing care					
Blood transfusion (days per week)					
0–2	125	87	69.6	1.00	
≥3	71	60	84.5	2.38	1.13–5.03
Resuscitation (days per week)					
0–2	148	104	70.3	1.00	
≥3	48	43	89.6	3.64	1.35–9.80
Preventive measures					
Use disposable gloves when exposed to contaminated items					
Sometimes/never	132	93	70.5	1.00	
Always	64	54	84.4	2.27	1.05–4.89
Segregate contaminated cotton, gauze and put in infectious-waste container					
Sometimes/never	63	41	65.1	1.00	
Always	133	106	79.7	2.11	1.08–4.11
Follow procedures whenever exposed					
To needlestick injury					
Sometimes/never	54	34	63.0	1.00	
Always	142	113	79.6	2.29	1.15–4.56
Support from hospital					
Allocated budget for HBV vaccination					
Inadequate	129	90	69.8	1.00	
Adequate	67	57	85.1	2.47	1.14–5.33

CI=Confidence interval; HBV=Hepatitis B virus; OR=Odds ratio

In the multivariate analysis, all variables with a p value of ≥0.05 in the 2×2 univariate analysis and biological plausibility were simultaneously analyzed by multiple logistic regressions. After adjustment for all other variables in the model, the results indicated that three variables were statistically associated with acceptance of HBV vaccination: history of accidental exposure to blood or blood products (adjusted odds ratio [OR] 4.76, 95% confidence interval [CI] 1.61–14.09), having acceptable knowledge of HBV infection (adjusted OR 3.45, 95%CI 1.03–11.54), and allocating an adequate budget for HBV vaccination (adjusted OR 3.07, 95% CI 1.19–7.92) (Table 6).

**Table 6:** Crude and adjusted odds ratios from multiple logistic regression analysis of factors relating to acceptance of HBV vaccination among 196 nursing students

Variable	Crude		Adjusted*	
	OR	95% CI	OR	95% CI
Accidental exposure to blood or blood products					
Never	1.00		1.00	
Ever	4.54	1.69–12.16	4.76	1.61–14.09
Knowledge about HBV infection					
Unacceptable	1.00		1.00	
Acceptable	4.06	1.37–12.04	3.45	1.03–11.54
Allocated budget for HBV vaccination					
Inadequate	1.00		1.00	
Adequate	2.47	1.14–5.33	3.07	1.19–7.92

*Adjusted for age, checking of HBV, history of accidentally exposure to blood or blood products, knowledge of HBV infection, nursing care, preventive measures, and level of allocated budget for HBV vaccination from the hospital; CI=Confidence interval; HBV=Hepatitis B virus; OR=Odds ratio

## DISCUSSION

The coverage of hepatitis B vaccination among HCWs is an important public-health issue. Vaccination not only prevents vaccine-preventable diseases, but also decreases the burden on the government. In this study, the overall acceptance of HBV vaccination among 196 nursing students was 75.0%; of these, 37.2% were completely vaccinated, and 37.8% were partially vaccinated. The overall acceptance rate of vaccination was comparable with nursing students in Taiwan, at 79.2% (95/120) (17). However, the complete vaccination rate was significantly lower (37.2% vs 69.2%). Two possible reasons were: (a) the differences in support policies in each hospital, and (b) a better socioeconomic status and follow-up system, so that a higher proportion of those who missed the scheduled vaccination were encouraged to receive fee-for-service vaccine (18). At several hospitals in many countries, all employees are offered free HBV vaccination (19-21). This suggests the need for further improvement in vaccination policies of hospitals with high levels of risk for their health personnel.

Multiple logistic regression analysis revealed that, after controlling for all other variables in the model, only three variables were significantly related to acceptance of HBV vaccination: history of accidental exposure to blood or blood products, acceptable knowledge about HBV infection, and adequate budget for HBV vaccination. The explanation was that there were partially correlations among predictor variables. Thus, beyond what non-significant predictors share with significant predictors, they did not account for any of the variance in the acceptance of HBV vaccination. For example, a student nurse has a higher level of knowledge simply because she spends more years of service as a nurse and is consequently more frequently exposed to blood and blood products. Her acceptable knowledge about HBV infection and availability of HBV vaccines in the hospital enable the nursing student in deciding to accept vaccination.

This study found that the odds of acceptance of HBV vaccination among nursing students accidentally exposed to blood or blood products was 4.76 times higher than for those not accidentally exposed. One possible explanation was that they might be more conscious about HBV infection and its prevention after exposure to patient's blood. Accidental exposure might change mindset of nursing students towards acceptance of HBV vaccination. This study confirmed the findings of Techapetpibul *et al*. (21) who reported that exposure to patient blood was associated with acceptance of HBV vaccination by nurses.

The findings also revealed that the odds of acceptance of HBV vaccination for the nursing students with ‘acceptable knowledge of HBV infection’ was 3.45 times higher than ‘unacceptable in knowledge’. It might be that knowledge of HBV infection and HBV vaccination resulted in positive attitudes among the nursing students and sustained their beliefs in the safety and efficacy of vaccine. These ‘acceptable knowledge’ respondents probably believed the information and opinions provided by their teachers, other nurses, and co-workers, or from their physicians. The result was consistent with the findings of McGrane and Staines who reported that obtaining information relating to the benefits of vaccine from an occupational health physician or from a nurse was a significant factor in acceptance of vaccine (22). Bradley and Kristi found that acceptance of HBV vaccine was strongly related to knowledge of HBV disease and HBV vaccination (23). However, in our study, 78.1% (153/196) of the nursing students were at an ‘unacceptable’ level regarding knowledge about HBV infection and vaccination.

The present study also found that the odds of acceptance of HBV vaccination for the nursing students who perceived that the hospital allocated an adequate budget for HBV vaccination were 3.07 times higher than those who perceived inadequate budgetary support. This might be due to the cost of vaccine such that the nursing students could not afford to buy the vaccine themselves. Mostly, in the study hospital, stocks were limited, and not every student was vaccinated on time or sometimes missed a dose due to a vaccine shortage. The result confirmed the finding of McGrane and Staines who reported that one factor that had a strong positive influence on nurses’ deciding to be vaccinated was the provision of free vaccine to HCWs (22).

This study had three main limitations: (a) although the study was conducted in an accredited teaching hospital in Pakistan, the results may not be generalizable to nursing students in other institutions in other parts of the country; (b) the study relied on self-reported data. However, self-reported data have been shown to have an acceptable sensitivity and specificity when investigating the vaccine coverage in the general population (24); and (c) the design of the study being cross-sectional, the results cannot be used for establishing a causal relationship. A follow-up study overcome this limitation.

In conclusion, the acceptance rate of HBV vaccination among nursing students was still low. All nursing students should be required to be vaccinated with hepatitis B vaccine prior to entry into clinical/practicum nursing, with support from health institutions. Follow-up systems, counselling about HBV, and effective intervention programmes designed to increase awareness relating to HBV infection, the procedures of knowledge and adhering to universally-accepted precautions, are also needed.

## ACKNOWLEDGEMENTS

The authors are grateful to all nursing students who participated in this study and to Dr. Elahi Bukhsh, Medical Superintendent of Bolan Medical Complex Hospital, Quetta, Pakistan, and Zaheer Aman Mengal, for their kind support during data collection.
